# Plasma Low-Density Lipoprotein Cholesterol Correlates With Heart Function in Individuals With Type 2 Diabetes Mellitus: A Cross-Sectional Study

**DOI:** 10.3389/fendo.2019.00234

**Published:** 2019-04-11

**Authors:** Po-Chung Cheng, Shang-Ren Hsu, Jung-Chi Li, Ching-Pei Chen, Szu-Chi Chien, Shih-Te Tu, Yun-Chung Cheng, Yu-Hsiu Liu, Jeng-Fu Kuo

**Affiliations:** ^1^Division of Endocrinology and Metabolism, Department of Internal Medicine, Changhua Christian Hospital, Changhua, Taiwan; ^2^Division of Cardiology, Department of Internal Medicine, Changhua Christian Hospital, Changhua, Taiwan; ^3^Department of Radiology, Taichung Veterans General Hospital, Taichung, Taiwan; ^4^Department of Accounting and Information Systems, National Taichung University of Science and Technology, Taichung, Taiwan

**Keywords:** type 2 diabetes mellitus, hyperlipidemia, low-density lipoprotein cholesterol, heart function, left ventricular ejection fraction, heart failure

## Abstract

**Background:** Heart failure is a frequent complication of type 2 diabetes mellitus (T2DM). Plasma cholesterol, particularly the proatherogenic low-density lipoprotein (LDL) cholesterol, impairs heart function by promoting atheroma formation and ventricular dysfunction. Considering the established effect of cholesterol on the cardiovascular system, we hypothesized that plasma LDL cholesterol may influence left ventricular function in individuals with T2DM.

**Methods:** This cross-sectional study was conducted at a tertiary care hospital in Taiwan. Enrollment criteria were patients exceeding 21 years of age with T2DM who received antidiabetic and cholesterol-lowering medications. Candidates were excluded if they had heart failure, acute cardiovascular events, or familial hypercholesterolemia. Participants received blood sampling for plasma lipids after a 12-h fast, followed by transthoracic echocardiography in the cardiology clinic.

**Results:** The study enrolled 118 participants who were divided into two groups according to their plasma LDL cholesterol levels. Demographic characteristics including age (69.7 vs. 66.9 years, *P* = 0.159), body mass index (26.2 vs. 25.9 kg/m^2^, *P* = 0.66), diabetes duration (5.4 vs. 5.1 years, *P* = 0.48), hemoglobin A_1c_ (7.2 vs. 7.5%, *P* = 0.225), and systolic blood pressure (129 vs. 130 mm Hg, *P* = 0.735) were similar between these groups. Moreover, all participants received similar antihypertensive medications. Participants with lower plasma LDL cholesterol levels had better heart function, as measured by the left ventricular ejection fraction (LVEF), than patients with higher LDL cholesterol levels (58.0 vs. 50.5%, *P* = 0.022). Multivariate regression analysis also showed an inverse correlation between plasma LDL cholesterol and left ventricular function (β coefficient: −0.110, *P* = 0.024).

**Conclusion:** This study observed an inverse correlation between plasma LDL cholesterol and heart function in individuals with T2DM. Patients with higher levels of plasma LDL cholesterol had worse left ventricular function. Therefore, plasma LDL cholesterol may be a modifiable risk factor of heart failure in diabetes, but prospective studies are necessary to confirm this finding.

## Introduction

Type 2 diabetes mellitus (T2DM) is a metabolic disease that affects a considerable number of patients worldwide (1). Among diabetic individuals, cardiovascular disease (CVD) is the leading cause of morbidity and mortality (2). Chronic hyperglycemia is associated with vascular dysfunction (3), oxidative stress (4), and proinflammatory cytokines that impair organ function (5). Moreover, patients with T2DM often have concomitant insulin resistance, which aggravates the severity of CVD (6).

The sympathoadrenal system has been implicated in the development of insulin resistance, which has detrimental effects on the cardiovascular system (7–9). Insulin resistance in diabetes is related to both glucose intolerance and impaired fatty acid metabolism (10). Indeed, the intimate relationship between fatty acid and glucose in the Randle cycle has strong implications for its role in the development of diabetes (11). Once insulin resistance has developed, it affects heart function through the activation of protein kinase C (12). Overall, hyperglycemia, dyslipidemia, and hypertensive cardiovascular disease contribute to heart failure in diabetic patients (13).

In the context of dyslipidemia, the low-density lipoprotein (LDL) cholesterol may impair heart function through several mechanisms. Besides its role as a proatherogenic lipoprotein, LDL cholesterol triggers the release of proinflammatory cytokines (14). Oxidized LDL cholesterol also promotes arterial intimal thickening to reduce myocardial blood flow (15). Although a link between plasma cholesterol and clinical outcome in heart failure has been reported (16), there is currently insufficient information about the influence of plasma LDL cholesterol on heart function.

Considering the link between plasma cholesterol and cardiovascular outcome, we hypothesized that plasma LDL cholesterol may influence heart function in diabetic patients. This study investigated the relationship between plasma LDL cholesterol levels and left ventricular function in individuals with T2DM. The influence of other plasma lipids such as triglycerides (TG) and high-density lipoprotein (HDL) cholesterol on heart function will also be evaluated.

## Materials and Methods

### Participant Selection

This cross-sectional study was conducted at Changhua Christian Hospital, a tertiary care hospital in Taiwan. Patients visiting the cardiology clinic between January 2016 and December 2017 were assessed for eligibility. Enrollment criteria were diabetic patients over 21 years of age who received antidiabetic and cholesterol-lowering medications. Candidates must be able to comply with transthoracic echocardiography for enrollment. Patients were excluded if they had previously been diagnosed with heart failure. Moreover, individuals with acute cardiovascular events, congenital heart disease, familial hypercholesterolemia, or chronic kidney disease were ineligible.

### Ethics Approval

This study was carried out in accordance with the World Medical Association's Declaration of Helsinki. The study was approved by the Institutional Review Board of Changhua Christian Hospital (CCH IRB No. 181103). All participants provided written informed consent to receive blood tests and transthoracic echocardiography in accordance with the Declaration of Helsinki.

### Laboratory Evaluation

Participants received blood tests for plasma TG, LDL cholesterol, HDL cholesterol, and glycated hemoglobin A_1c_ (HbA_1c_) after a 12-h fast. Blood samples were sent to the central laboratory within 1 h and assayed by Beckman Coulter UniCel DxC 800 Synchron™ Clinical Systems. Specifically, plasma LDL cholesterol was measured by the timed-endpoint method using a commercial polyanion solution. Analytical precision was within 1.7, 3.0, and 7.5 ml/dL for HDL cholesterol, LDL cholesterol and TG, respectively.

### Echocardiographic Assessment

Sonographers at the cardiology clinic performed transthoracic echocardiography using Canon Medical Systems' Aplio™ 300 CV Platinum system. Echocardiographic measurements were performed according to current guidelines (17). Specifically, the left atrial diameter, left ventricular end systolic diameter (LVESD) and end diastolic diameter (LVEDD) were measured in the parasternal long axis view. The right ventricular diameter was measured in the apical four-chamber view. E and A velocities from mitral valve inflow were used to calculate the E/A ratio. The left ventricular ejection fraction (LVEF) was derived from the modified Quinones equation (18).

### Statistical Analysis

Participants were divided into two groups according to their plasma LDL cholesterol levels. Power analysis indicated that a sample size of 48 participants per group was necessary to detect a significant difference in left ventricular function with 80% statistical power. The Kolmogorov-Smirnov test was used to confirm the normal distribution of clinical variables in this study (D = 0.11807, *P* = 0.06859). Demographic characteristics between groups were compared using Student's independent *t*-test for continuous variables and Pearson's χ^2^-test for categorical variables. Echocardiographic parameters were compared using Student's independent *t*-test. Moreover, multivariate regression analysis was used to examine the relationship between left ventricular function and plasma lipids. Statistical analysis was performed using IBM SPSS version 22.0 (IBM SPSS Statistics for Windows. Armonk, NY, USA) with a two-tailed *P* < 0.05 indicating statistical significance.

## Results

**Figure 1:**
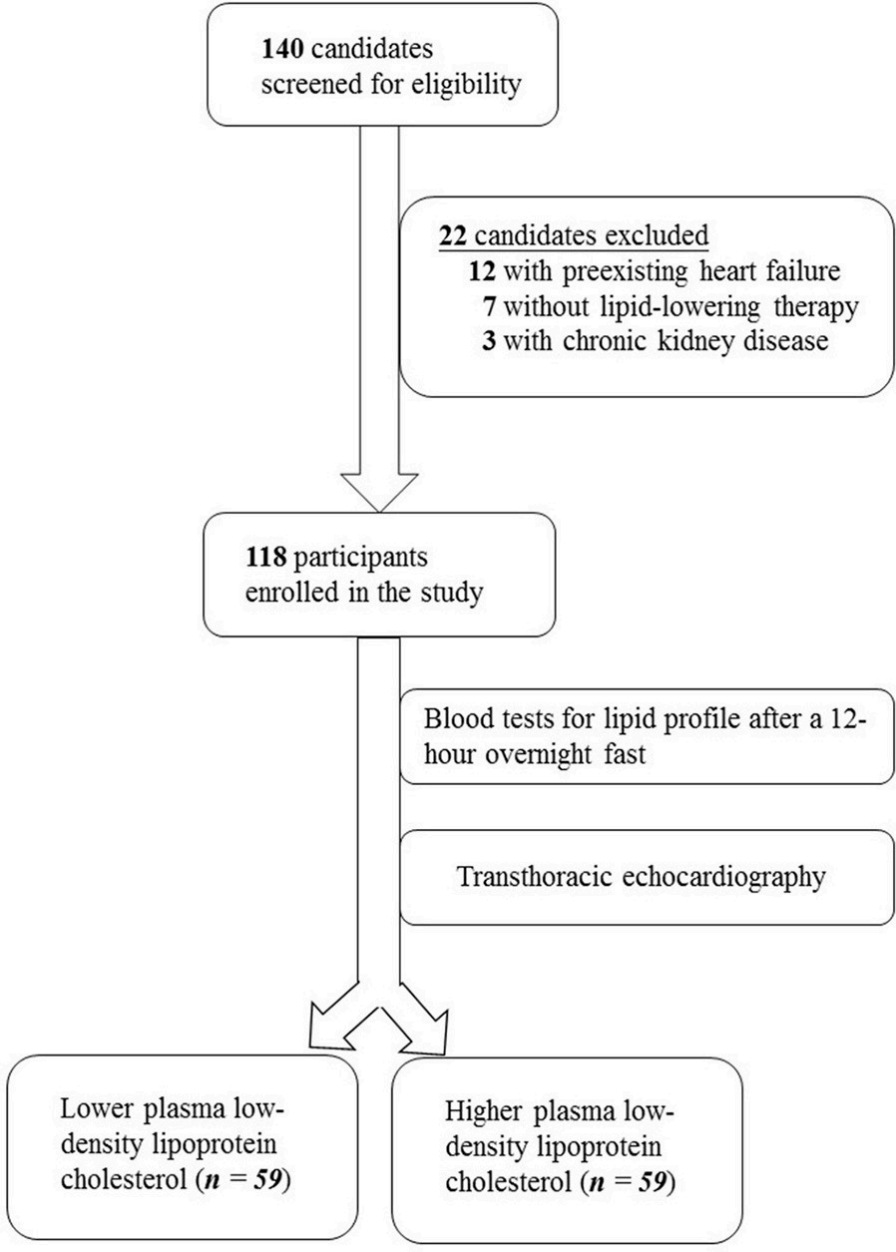
Enrollment protocol of the study.

The study screened 140 individuals for eligibility. Twelve individuals were excluded due to heart failure, seven patients did not receive cholesterol-lowering medications and were thus ineligible, and three candidates with chronic kidney disease were excluded. The enrollment process is illustrated in Figure 1.

### Demographic Characteristics of Participants

The study enrolled a total of 118 participants who were divided into two groups according to their plasma LDL cholesterol levels. As summarized in Table 1, demographic characteristics including age (69.7 vs. 66.9 years, *P* = 0.159), female sex (45.8 vs. 42.4%, *P* = 0.711), body mass index (26.2 vs. 25.9 kg/m^2^, *P* = 0.66), diabetes duration (5.4 vs. 5.1 years, *P* = 0.48), plasma TG level (140 vs. 141 mg/dL, *P* = 0.945), plasma HDL cholesterol level (36.1 vs. 38.8 mg/dL, *P* = 0.129), serum HbA_1c_ (7.2 vs. 7.5%, *P* = 0.225), and systolic blood pressure (129 vs. 130 mm Hg, *P* = 0.735) were similar between these groups. Comparable proportions of participants in both groups received beta blockers (62.7 vs. 69.5%, *P* = 0.437), calcium channel blockers (44.1 vs. 40.0%, *P* = 0.653), angiotensin converting enzyme inhibitors or angiotensin receptor blockers (66.1 vs. 55.9%, *P* = 0.258), and diuretics (18.6 vs. 23.7%, *P* = 0.499). All participants received cholesterol-lowering medications. The mean plasma LDL cholesterol level was significantly different between these groups (63.0 vs. 112 mg/dL, *P* < 0.001), which formed the basis of this clinical investigation.

**Table 1:** Demographic features of the participants.

**Parameters**	**Lower plasma LDL cholesterol (*n* = 59)**	**Higher plasma LDL cholesterol (*n* = 59)**	***P-*value**
Age (years)	69.7 ± 1.30 (67.1–72.3)	66.9 ± 1.45 (64.0–69.8)	0.159
Sex (Female)	27 (45.8%)	25 (42.4%)	0.711
Serum HbA_1c_ (%)	7.2 ± 0.15 (6.9–7.5)	7.5 ± 0.21 (7.1–7.9)	0.225
Body mass index (kg/m^2^)	26.2 ± 0.42 (25.4–27.0)	25.9 ± 0.52 (24.9–26.9)	0.660
Diabetes duration (years)	5.4 ± 0.24 (4.9–5.8)	5.1 ± 0.23 (4.7–5.6)	0.48
Systolic blood pressure (mm Hg)	129 ± 2.24 (124–133)	130 ± 2.62 (125–135)	0.735
Plasma triglycerides (mg/dL)	140 ± 12.2 (115–164)	141± 11.0 (119–163)	0.945
Plasma HDL cholesterol (mg/dL)	36.1 ± 1.35 (33.4–38.8)	38.8 ± 1.14 (36.5–41.1)	0.129
Plasma LDL cholesterol (mg/dL)	63.0 ± 1.48 (60.0–66.0)	112 ± 3.78 (105–120)	<0.001
Beta blocker (%)	37 (62.7%)	41 (69.5%)	0.437
Calcium channel blocker (%)	26 (44.1%)	23 (40.0%)	0.653
ACEI/ARB (%)	39 (66.1%)	33 (55.9%)	0.258
Diuretics (%)	11 (18.6%)	14 (23.7%)	0.499

*Data are expressed as means with standard error of the mean (95% confidence interval) for continuous variables and number (%) for categorical variables. Variables are compared between groups using Student's independent t-test for continuous data and Pearson's χ^2^-test for categorical data. HbA_1c_, glycosylated hemoglobin A_1c_; HDL, high-density lipoprotein; LDL, Low-density lipoprotein; mg/dL, milligrams per deciliter; mm Hg, millimeters of mercury; ACEI, angiotensin converting enzyme inhibitor; ARB, angiotensin receptor blocker*.

### Correlation Between Plasma LDL Cholesterol and Left Ventricular Function

As shown in Table 2, participants with lower plasma LDL cholesterol levels had better heart function, as measured by the LVEF, than patients with higher LDL cholesterol levels (58.0 vs. 50.5%, *P* = 0.022). These groups had similar left atrial diameter (43.2 vs. 43.3 mm, *P* = 0.913), right ventricular diameter (25.3 vs. 25.1 mm, *P* = 0.81), LVESD (31.5 vs. 33.0 mm, *P* = 0.43), LVEDD (48.7 vs. 49.7 mm, *P* = 0.476), and E/A ratio (0.96 vs. 0.93, *P* = 0.57).

**Table 2:** Association between plasma low-density lipoprotein cholesterol and left ventricular function.

**Parameters**	**Lower plasma LDL cholesterol (*n* = 59)**	**Higher plasma LDL cholesterol (*n* = 59)**	***P*-value**
Left ventricular ejection fraction (%)	58.0 ± 2.24 (53.5–62.5)	50.5 ± 2.38 (45.7–55.2)	0.022
Left atrial diameter (mm)	43.2 ± 0.80 (41.6–44.8)	43.3 ± 1.15 (41.0–45.6)	0.913
Left ventricular end diastolic diameter (mm)	48.7 ± 0.768 (47.2–50.2)	49.7 ± 1.11 (47.5–51.9)	0.476
Right ventricular diameter (mm)	25.3 ± 0.624 (24.1–26.6)	25.1 ± 0.554 (24.0–26.2)	0.81
Left ventricular end systolic diameter (mm)	31.5 ± 1.13 (29.3–33.8)	33.0 ± 1.46 (30.1–35.9)	0.43
E/A ratio	0.96 ± 0.04 (0.88–1.0)	0.93 ± 0.035 (0.86–1.0)	0.57

*Data are expressed as means with standard error of the mean (95% confidence interval) for continuous variables. Variables are compared between groups using Student's independent t-test. LDL, low-density lipoprotein; mm, millimeters*.

### Multivariate Regression Analysis of LVEF Determinants

In Table 3, multivariate regression analysis revealed that plasma LDL cholesterol was inversely correlated with left ventricular function (β coefficient: −0.110, *P* = 0.024). In contrast, other plasma lipids such as TG (β coefficient: 0.026, *P* = 0.142) and HDL cholesterol (β coefficient: 0.182, *P* = 0.278) had limited correlation with LVEF. The structural parameter LVEDD was shown to be correlated with left ventricular function (β coefficient: −1.085, *P* = 0.001).

**Table 3:** Multivariate regression analysis of parameters associated with left ventricular ejection fraction.

**Parameters**	**β coefficient**	***P*-value**
Age (years)	0.021	0.893
Body mass index (kg/m^2^)	0.129	0.765
Diabetes duration (years)	−0.376	0.660
Plasma LDL cholesterol (mg/dL)	−0.108	0.024
Plasma HDL cholesterol (mg/dL)	0.179	0.295
Plasma triglycerides (mg/dL)	0.025	0.170
Serum HbA_1c_ (%)	−0.811	0.484
Systolic blood pressure (mm Hg)	−0.012	0.894
Left atrial diameter (mm)	0.039	0.863
Left ventricular diastolic diameter (mm)	−1.096	0.001

*HbA_1c_, glycosylated hemoglobin A_1c_; HDL, high-density lipoprotein; LDL, Low-density lipoprotein; mm, millimeters; mg/dL, milligrams per deciliter; mm Hg, millimeters of mercury*.

## Discussion

This study revealed an inverse relationship between plasma LDL cholesterol levels and heart function in patients with T2DM. Specifically, patients with lower plasma LDL cholesterol levels had better LVEF than participants with higher LDL cholesterol levels. Moreover, multivariate regression analysis showed a link between left ventricular function and plasma LDL cholesterol. To our knowledge, this is the first study to investigate the association between plasma LDL cholesterol and heart function in patients with diabetes.

Several physiologic mechanisms may explain the link between plasma LDL cholesterol and heart function. First, LDL cholesterol is a recognized risk factor of atherosclerotic heart disease and heart failure mortality (19, 20). Furthermore, investigators have demonstrated that insulin resistance can decrease myocardial glucose uptake to impair heart function (21). Excessive lipid metabolites also lead to structural abnormalities such as myocardial fibrosis (22). As mentioned previously, a synergistic effect between hyperglycemia, dyslipidemia, and hypertensive cardiovascular disease leads to heart failure in patients with T2DM.

This study excluded candidates with heart failure to avoid the cholesterol paradox. Individuals with terminal heart failure paradoxically fared better with higher plasma cholesterol levels (23). This phenomenon may be attributable to heart failure induced cachexia, which reduces plasma cholesterol levels (24). Researchers also suggest that the presence of CVD can modify the relationship between plasma cholesterol and heart function (16).

The inverse relationship between plasma LDL cholesterol levels and heart function has clinical implications. Clinical trials have shown that cholesterol-lowering therapy in diabetic patients can reduce CVD mortality (25). Our study suggests that cholesterol-lowering intervention may also preserve heart function in individuals with T2DM. Indeed, a recent study suggests that the lipid-lowering effect of insulin can improve heart function in elderly individuals with diabetes (26). In addition, medical nutrition therapy that targets LDL cholesterol may attenuate the development of heart failure.

This study provides novel information about the relationship between plasma LDL cholesterol and left ventricular function. Echocardiographic measurements were verified by three cardiologists to lessen the inter-operator variability (*F* = 2.1, *P* = 0.22). Participants received blood tests at the same medical center, which helps to reduce variability in laboratory techniques. Participants in both groups also received similar antihypertensive medications to reduce the potential confounding effects of these medications on heart function.

However, this study also has limitations. Since sonographic examination is technique-dependent (27), the accuracy of LVEF measurement depends on the sonographer's experience. Moreover, dietary intake of omega-3 polyunsaturated fatty acids can influence left ventricular function (28). Thirdly, antidiabetic medications such as sodium glucose cotransporter 2 inhibitors can modify left ventricular contractility (29). Obesity is also related to heart failure, but abdominal circumference was not included in the analysis. The study is also limited by its non-randomized design, relatively small sample size, and cross-sectional design. Therefore, prospective studies with a larger sample size are required to confirm its findings.

In conclusion, there is an inverse correlation between plasma LDL cholesterol and heart function in people with diabetes. Participants with higher levels of plasma LDL cholesterol had worse LVEF than patients with lower LDL cholesterol levels. Therefore, this study supports the hypothesis that plasma LDL cholesterol may influence heart function in diabetic patients, but prospective studies are necessary to confirm this finding.

## Data Availability

The raw data supporting the conclusions of this manuscript will be made available by the authors, without undue reservation, to any qualified researcher.

The research dataset is available as Supplementary Table 1.

## Ethics Statement

This study was carried out in accordance with the World Medical Association's Declaration of Helsinki. The study was approved by the Institutional Review Board of Changhua Christian Hospital (CCH IRB No. 181103). All participants provided written informed consent to receive blood tests and transthoracic echocardiography in accordance with the Declaration of Helsinki.

## Author Contributions

P-CC, S-RH, J-CL, C-PC, S-CC, S-TT, and J-FK conceived and designed the experiments, performed the experiments, contributed reagents, materials, analysis tools, and wrote the manuscript. Y-CC and Y-HL analyzed the data, prepared figures and tables, and wrote the manuscript. Y-HL is a qualified statistician who verified the statistical methods in this study. P-CC and Y-CC contributed equally to the study as first authors. All authors have reviewed and approved of the manuscript to be submitted.

### Conflict of Interest Statement

The authors declare that the research was conducted in the absence of any commercial or financial relationships that could be construed as a potential conflict of interest.
